# *Mycobacterium tuberculosis*-specific CD4 T cells expressing CD153 inversely associate with bacterial load and disease severity in human tuberculosis

**DOI:** 10.1038/s41385-020-0322-6

**Published:** 2020-07-16

**Authors:** Elsa Du Bruyn, Sheena Ruzive, Cecilia S. Lindestam Arlehamn, Alessandro Sette, Alan Sher, Daniel L. Barber, Robert J. Wilkinson, Catherine Riou

**Affiliations:** 1 Wellcome Centre for Infectious Disease Research in Africa, Institute of Infectious Disease and Molecular Medicine, University of Cape Town, Observatory, Cape Town, 7925 South Africa; 2 Division of Vaccine Discovery, La Jolla Institute for Immunology, La Jolla, CA USA; 3 Department of Medicine, University of California San Diego, La Jolla, CA USA; 4 Immunobiology Section, Laboratory of Parasitic Diseases, National Institute of Allergy and Infectious Diseases, National Institutes of Health, Bethesda, MD USA; 5 T Lymphocyte Biology Section, Laboratory of Parasitic Diseases, National Institute of Allergy and Infectious Diseases, National Institutes of Health, Bethesda, MD USA; 6 Department of Infectious Diseases, Imperial College London, London, W2 1PG UK; 7 Department of Medicine, University of Cape Town, Observatory, Cape Town, 7925 South Africa; 8 The Francis Crick Institute, London, NW1 1AT UK; 9 Division of Medical Virology, Department of Pathology, University of Cape Town, Cape Town, South Africa

## Abstract

Recent data from mice and non-human primate models of tuberculosis suggested that CD153, a TNF super family member, plays an important role in *Mycobacterium tuberculosis* (Mtb) control. However, this molecule has not been comprehensively evaluated in humans. Here, we show that the proportion of Mtb-specific CD4 T cells expressing CD153 was significantly reduced in active TB patients compared to latently infected persons. Importantly, the CD153+ Mtb-specific CD4 response inversely correlated with lung bacterial load, inferred by Xpert cycle threshold, irrespective of HIV status. Antitubercular treatment partially restored CD153 expression on Mtb-specific CD4 T cells. This is the first report of a subset of Mtb-specific CD4 T cells showing strong negative correlation with bacterial burden. Building on substantial evidence from animal models implicating CD153 as a mediator of host protection, our findings suggest it may play a similar role in humans and its measurement may be useful to evaluate TB vaccine efficacy.

## Introduction

Tuberculosis (TB) remains the leading cause of death by infection worldwide with nearly 10 million new cases and about 1.5 million TB-related deaths in 2018.^1^ CD4 T-cell responses are critical for control of *Mycobacterium tuberculosis* (Mtb) infection, and HIV-infection-associated CD4 T-cell depletion greatly increases susceptibility to developing TB disease.

Although IFNγ producing CD4 T cells are key components of the immune response against Mtb, these cells alone are insufficient to provide protection.^2,3^ A complex array of T cells are implicated in the immune response to TB, but their relative contribution to protection remains undefined;^4–6^ and the precise characteristics of protective Mtb-specific CD4 T cells remain elusive, hindering the development of new vaccines. A study in mice showed that CD153 (a molecule belonging to the tumor necrosis factor (TNF) super family, also named CD30L or TNFSF8) expressed by CD4 T cells was necessary for Mtb control in the lung, independently of IFNγ.^7^ Moreover, complementary data from a non-human primate (NHP) Mtb infection model showed that the frequency of Mtb-specific CD4 T cells expressing CD153 inversely correlated with bacterial load in granulomas.^7^ Further evidence supporting a role for CD153 in Mtb protection arose from a recent study assessing the protective potential of intravenous BCG immunization.^8^ Darrah et al. reported that intravenous BCG vaccination, which showed superior protection to Mtb challenge compared to intradermal or aerosol route of vaccination in NHPs, induced a unique CD4 T cell response enriched with CD153 gene transcripts together with other genes associated with protection against TB, such as IFNγ and IL21R.^8,9^

Data on the role of CD153 in the anti-mycobacterial immune response in humans are scarce. We demonstrated, in a small number of persons (*n* = 16), that CD153 expression in Mtb-specific CD4 T cells was significantly lower in patients with active TB disease compared to latently infected persons.^7^ However, the relationship between CD153 and bacterial burden remained unexplored and the expression and regulation of CD153 in Mtb-specific CD4 responses in HIV-infected persons have not been investigated. The objective of this study was to (1) investigate whether any association exists between CD153-expressing Mtb-specific CD4 T cells and bacterial load in sputum of those with active TB, (2) define the effect of HIV coinfection on this cell subset, and (3) assess the impact of successful antitubercular therapy (ATT) on CD153 expression by CD4 T cells responding to Mtb.

## Results

### Study population

The clinical characteristics of participants are presented in Table 1. Participants (*n* = 137) were classified into four groups according to their HIV-1 and TB status: latent TB infection (LTBI)/HIV− (*n* = 35), LTBI/HIV+ (*n* = 32), aTB (active TB)/HIV− (*n* = 33), and aTB/HIV+ (*n* = 37). Median age was comparable between the four groups. IGRA values were comparable between the HIV-uninfected and HIV-infected LTBI groups (*P* = 0.14). Xpert MTB/RIF cycle threshold (Xpert C_T_) was higher in HIV-infected compared to HIV-uninfected patients (median: 21.7 vs 18.3, respectively, *P* = 0.02). HIV infection did not affect the level of plasma C-reactive protein (CRP) in aTB patients (medians: 76.5 µg/ml in HIV+ and 100 µg/ml in HIV−, *P* = 0.99). Finally, HIV-infected LTBI participants had a significantly lower plasma HIV-1 viral load (VL) and higher absolute CD4 count compared to the HIV-infected aTB group (median VL: <20 vs 9044 copies/ml, *P* = 0.01 and median CD4: 481 vs 273 cells/mm^3^, *P* = 0.0004, respectively). These differences are due to higher antiretroviral therapy (ART) usage in the LTBI group compared to the aTB group (78.1% vs 48.6%, respectively, *P* = 0.011). Follow-up blood samples were available for 28 HIV-uninfected participants and 32 for HIV-infected patients after the completion of standard ATT regimen at week 24.

**Table 1:** Clinical characteristics of study participants.

	LTBI/HIV−	LTBI/HIV+	aTB/HIV−	aTB/HIV+	*P* values^b^
*N*	35	32	33	37	
Age (year)^a^	31 (23–38)	34 (31–42)	30 (24–43)	37 (32–45)	
Gender (F/M)	19/16	24/8	8/25	11/26	
IGRA (IU/ml)^a,c^	6.6 (2.4–10)	3.2 (1.9–8.4)	nd	nd	0.14
CD4 (cells/mm^3^)^a^	nd	481 (358–700)	nd	273 (148–435)	**0.0004**
VL (mRNA copies/ml)^a^	na	<20 (<20–15,620)	na	9,044 (<20–94,600)	**0.01**
% on ART	na	78.1 %	na	48.6%	**0.011**
XPert C_T_ value^a,d^	na	na	18.3 (15.5–22)	21.7 (17.2–27.3)	**0.02**
CRP (µg/ml)^a^	1 (1–4)	3 (2–11.5)	100 (27.5–113)	76.5 (30–123)	**0.02**^e^/0.99^f^

*LTBI* latent tuberculosis infection, *aTB* active tuberculosis disease, *F* female, *M* male, *IGRA* Interferon Gamma Release Assay, *VL* HIV-1 viral load, *ART* antiretroviral treatment, *Xpert C*_*T*_ Xpert MTB/RIF cycle threshold, *CRP* C-reactive protein, *nd* not done, *na* not applicable.

The bold highlights statistical significance.

^a^Median and interquartile ranges.

^b^*P* values were calculated using a nonparametric Mann–Whitney test for all measurements except for ART usage where a chi-square test was applied.

^c^For IGRA, results are reported as (TB Ag-Nil). The cut-off point for positivity was 0.36 IU/ml.

^d^Xpert cycle threshold value for each participant was defined as the average value of the 5 probes used in the assay. Xpert C_T_ values were missing for two HIV-infected aTB participants.

^e^*P* value between the LTBI/HIV− and LTBI/HIV+ groups.

^f^*P* value between the aTB/HIV− and aTB/HIV+ groups.

### Measures of mycobacterial load in sputum

**Fig. 1:**
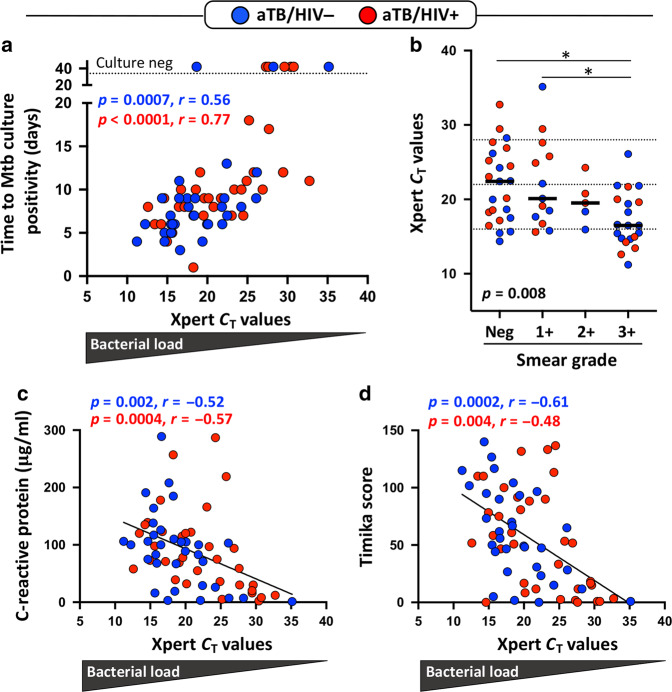
Sputum Xpert MTB/RIF cycle threshold (Xpert C_T_) values associates with other measures of Mtb Burden (time to Mtb culture positivity (TTP) and smear microscopy grade), systemic inflammation (CRP), and radiographic evidence of disease severity (Timika score). **a** Correlation between Xpert C_T_ values and time to Mtb culture positivity in HIV-uninfected (blue) and HIV-infected (red) participants. **b** Correlation between smear microscopy grade and Xpert C_T_ values. Horizontal lines represent the median values. **c**, **d** Correlations between Xpert C_T_ values and plasma C-reactive protein and Timika score on chest radiograph. The solid lines indicate the linear regression and correlations between measures were performed using a two-tailed nonparametric Spearman test.

Xpert C_T_ values correlated with time to Mtb culture positivity (TTP) both in HIV-uninfected and HIV-infected patients (*P* = 0.0007, *r* = 0.56 and *P* < 0.0001, *r* = 0.77, respectively) (Fig. 1a). The smear grade increased as the Xpert C_T_ values decreased (Fig. 1b, *P* = 0.008). These results are in accordance with previous publications.^10–12^ Furthermore, regardless of HIV status, we found that Xpert C_T_ values negatively correlated with plasma CRP levels (*P* = 0.002, *r* = −0.52 for HIV− and *P* = 0.0004, *r* = −0.57 for HIV+; Fig. 1c) and the Timika radiographic severity score (*P* = 0.0002, *r* = −0.61 for HIV− and *P* < 0.004, *r* = −0.48 for HIV+; Fig. 1d). Overall, these results show that the Xpert C_T_ value is a robust measure of Mtb load, correlating with radiographic evidence of TB disease severity, irrespective of the participant’s HIV status.

### CD153 expression in Mtb-specific CD4 T cell responses and its relationship with bacterial burden

**Fig. 2:**
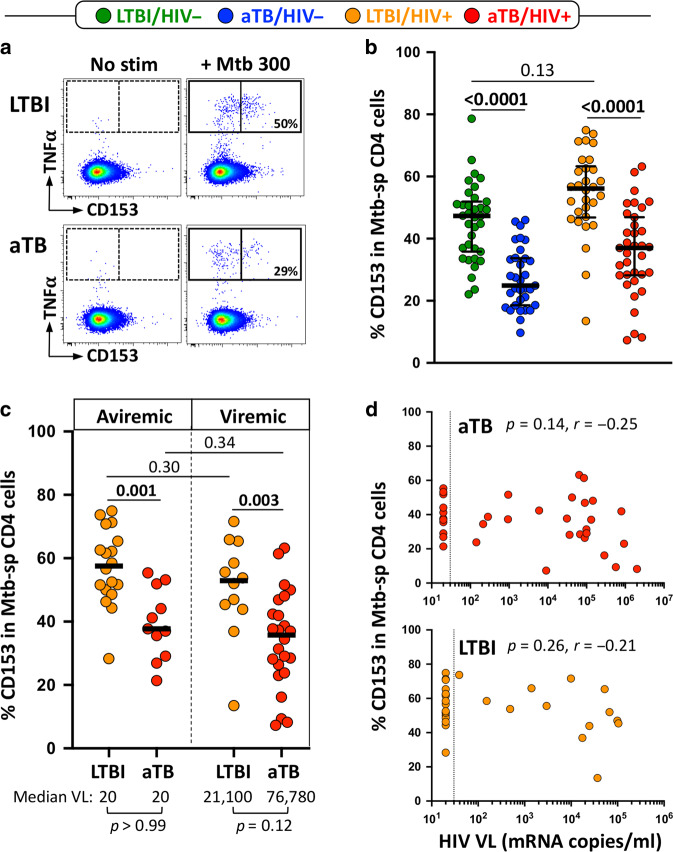
The proportion of Mtb300-specific CD4 T cells expressing CD153 is reduced in aTB compared to LTBI regardless of HIV status. **a** Representative flow cytometry plots showing the expression of CD153 in Mtb300-spcific CD4 T cells in one LTBI and one aTB participant. No stim = no stimulation; Mtb300 = stimulation with a pool of 300 Mtb-derived peptides. **b** Proportion of Mtb300-specific CD4 T cells expressing CD153 in LTBI/HIV− (*n* = 35, green), aTB/HIV− (*n* = 33, blue), LTBI/HIV+ (*n* = 30, orange), and aTB/HIV+ (*n* = 35, red). Error bars indicate medians and interquartile ranges. Statistical comparisons were performed using One-way ANOVA Kruskal–Wallis test with a Dunn’s multiple comparison test. CD153 expression was assessed only on Mtb300-specific response (i.e CD4 T cells expressing any measured cytokines) exhibiting more than 30 events. **c** CD153 expression in Mtb300-specific CD4 T cells in LTBI and aTB HIV-infected participants stratified based on plasma HIV viral load (aviremic vs viremic). Median VL for each subgroup is presented at the bottom of the graph. Statistical comparisons were performed using Mann–Whitney test. **d** Correlation between HIV viral load and the proportion of CD153+ Mtb300-specific CD4 T cells in the aTB (top) and LTBI (bottom) groups. Correlations were tested by a two-tailed nonparametric Spearman rank test.

While Th1 responses are necessary to control Mtb replication,^5^ the magnitude of the Mtb-specific CD4 T cell response did not associate with bacterial burden (Supplementary Fig. 1 and previously reported in ref. ^13^) suggesting that the quality rather than the quantity of Mtb-specific CD4 T cells are critical for Mtb control. The recent findings showing that (1) CD153 is an essential molecule for T cell-dependent control of Mtb in the mouse lung^7^ and (2) BCG-specific T cells induced in response to intravenous BCG immunization (mediating a durable protection against Mtb) are enriched in CD153 in non-human primates (NHP)^8^ prompted us to investigate the relationship between CD153 expression by Mtb-specific CD4 T cells and TB disease severity in humans. First, we compared CD153 expression by Mtb300-specific CD4 T cells in LTBI and aTB participants with or without HIV infection (Fig. 2a). The proportion Mtb300-specific CD4 T cells expressing CD153 was significantly decreased in aTB compared to LTBI in both HIV-uninfected participants (median: 30% vs 47%, respectively, *P* < 0.0001) and HIV-infected participants (37% vs 56%, *P* < 0.0001) (Fig. 2b). As there was a significant disparity in HIV VL between the LTBI and aTB groups (Table 1), we stratified participants into those who were virally suppressed (aviremic) and those who had an unsuppressed HIV VL (viremic) to define whether ongoing viral replication affects CD153 expression by Mtb-specific CD4 T cells (Fig. 2c). Our results show that, Mtb300-specific CD4 T cells expressing CD153 was decreased in aTB patients compared to LTBI, irrespective of their viremic status (*P* = 0.001 in those who are aviremic and *P* = 0.002 in viremic participants). Moreover, CD153+ Mtb300-specific CD4 T cells were not associated with HIV VL in the aTB group or in the LTBI group (*P* = 0.14, *r* = −0.25 and *P* = 0.26, *r* = −0.21, respectively) (Fig. 2d). Similar results were obtained when participants were stratified based on their CD4 count (≤300 or >300 cells/mm^3^) (Supplementary Fig. 2a). No association was found between Mtb300-specific CD4 T cells expressing CD153 and absolute CD4 count (Supplementary Fig. 2b). Overall, these data show that the presence of CD153-expressing CD4 T cells correlates with improved bacterial control; and that CD153 expression by Mtb300-responding CD4 T cells was not affected by HIV infection.

**Fig. 3:**
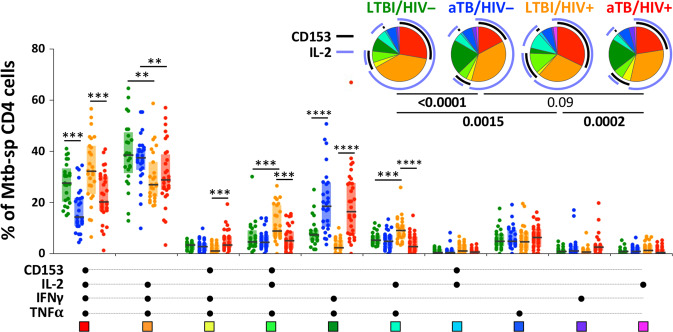
Polyfunctional Mtb300-specific CD4 T cells (i.e CD153+ IL-2+ IFNγ+ TNFα+) are selectively reduced in aTB participants compared to LTBI, regardless of HIV status. The *x*-axis displays each of the different response patterns, the composition of which is denoted with a dot for the presence of CD153, IL‐2, IFNγ, and TNFα. The proportion of each response pattern contributing to the total Mtb300-specific CD4 response per individual is shown. The median (gray bar) and interquartile ranges (box) are shown. Subsets accounting for <1% of the total Mtb300-specific CD4 T cell response are not displayed. Each response pattern is color‐coded, and data are summarized in the pie charts, where each pie slice represents the median contribution of each response pattern to the total Mtb300 response. A Wilcoxon rank-sum test was used to compare response patterns between groups (****P < 0.0001, ***P < 0.001, **P < 0.01). Statistical differences between pie charts were defined using a permutation test.

To further analyze the quality of Mtb CD4 responses, we defined the polyfunctional potential of Mtb300-specific CD4 T cells based on their capacity to coexpress CD153, IFNγ, TNFα, or IL-2. Figure 3 shows that CD153 expression was mainly restricted to a subset of highly polyfunctional CD4 T cells coproducing IFNγ, TNFα, and IL-2. This quadruple-positive subset was significantly reduced in patients with aTB compare to those latently infected (irrespective of HIV infection), while no change was observed for triple-producing cells making IFNγ, TNFα, and IL-2 but negative for CD153 (Fig. 3).

**Fig. 4:**
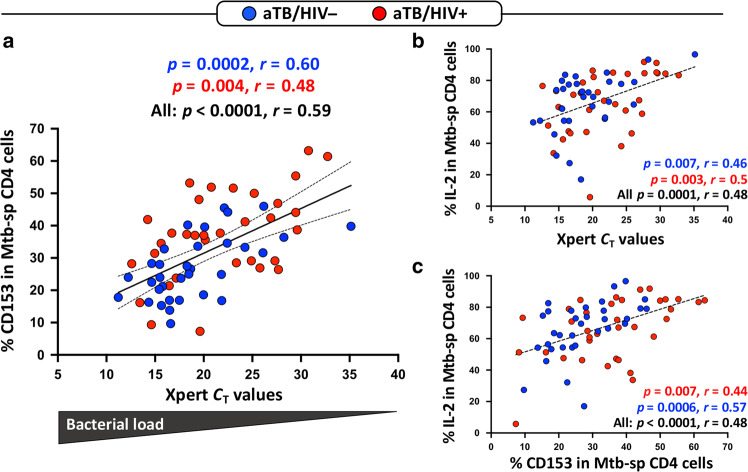
The proportion of CD153+ Mtb300-specific CD4 T cells inversely correlates with Mtb bacterial burden, irrespective of HIV status. **a** Correlation between Xpert C_T_ values and the proportion of CD153+ Mtb300-specific CD4 T cells in HIV-uninfected (blue) and HIV-infected (red) persons. Linear regression and 95% confidence band are depicted. **b** Correlation between Xpert C_T_ values and the proportion of IL-2+ Mtb300-specific CD4 T cells in HIV-uninfected and HIV-infected persons. **c** Correlation between the proportion of CD153+ Mtb300-specific CD4 T cells and the proportion of IL-2+ Mtb300-specific CD4 T cells in HIV-uninfected and HIV-infected persons. Correlations were tested by a two-tailed nonparametric Spearman rank test.

Importantly, the proportion Mtb300-specific CD4 T cells expressing CD153 positively associated with Xpert C_T_ values in both HIV-uninfected (*P* = 0.0002, *r* = 0.6) and HIV-infected patients (*P* = 0.004, *r* = 0.48) (Fig. 4a), showing that higher expression of CD153 denotes lower Mtb bacterial burden. This result is in keeping with data obtained from the NHP model of Mtb infection.^7^ Moreover, CD153-expressing Mtb300-specific CD4 T cells inversely correlated with CRP level (*P* = 0.005, *r* = −0.34), Timika radiographic severity (*P* = 0.016, *r* = −0.29) and time to Mtb culture positivity (*P* = 0.019, *r* = 0.28) when all aTB participants were combined (Supplementary Fig. 3). We also found association between the proportion Mtb300-specific CD4 T cells expressing IL-2 and Xpert C_T_ values (Fig. 4b). In fact, expression of CD153 on Mtb300-specifc T cells correlated positively with that of IL-2, irrespective of HIV status (*P* = 0.0006, *r* = 0.57 for HIV− and *P* = 0.007, *r* = 0.44 for HIV+), suggesting that both genes could share a common regulatory network (Fig. 4c).

### Phenotypic characteristics of polyfunctional Mtb300-specific CD4 T expressing CD153

**Fig. 5:**
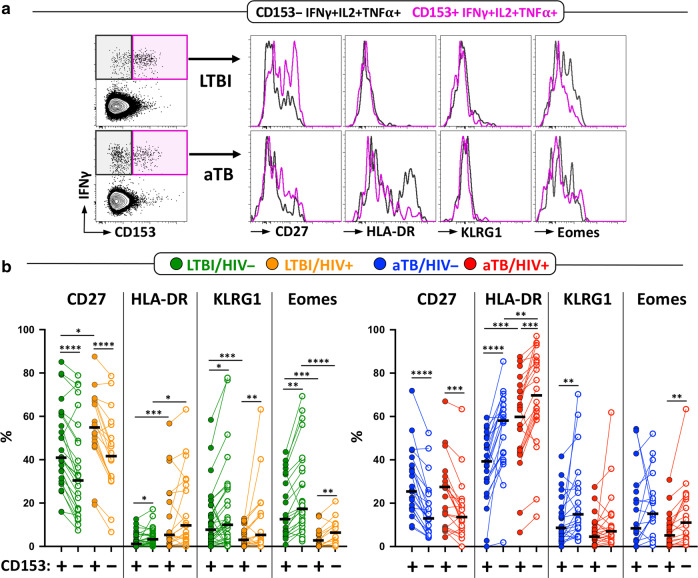
CD153+ polyfunctional Mtb300-specific CD4 T cells are phenotypically less activated and differentiated than CD153− polyfunctional cells. **a** Representative flow cytometry overlay plots of CD27, HLA-DR, KLRG1, and Eomes expression in CD153+ IL-2+ IFNγ + TNFα+ (pink) and CD153− IL-2+ IFNγ+ TNFα+ (black) Mtb300-specific CD4 T cells in one LTBI and one aTB participant. **b** Summary graph of the expression of CD27, HLA-DR, KLRG1, and Eomes in CD153+ IL-2+ IFNγ+ TNFα+ [CD153+] and CD153- IL-2+ IFNγ+ TNFα+ [CD153−] Mtb300-specific CD4 T cells in LTBI (left) and aTB (right) participants. Only CD153+ IL-2+ IFNγ+ TNFα+ and CD153- IL-2+ IFNγ+ TNFα+ subpopulations with more than 30 events were considered for phenotyping. Statistical comparisons were performed using a paired nonparametric Wilcoxon test. (****P < 0.0001, ***P < 0.001, **P < 0.01, *P < 0.05).

To better understand the specific phenotypic attributes of polyfunctional Mtb300-specific CD4 T cells expressing CD153, we compared the expression of CD27 (memory marker), HLA-DR (activation marker), KLRG1 (coinhibitory molecule),^14^ and Eomes (Transcription factor)^15^ in CD153+ IFNγ+ TNFα+ IL-2+ and CD153− IFNγ+ TNFα+ IL-2+ Mtb300-specific CD4 T cells in our four study groups (Fig. 5a). During LTBI, CD153+ IFNγ+ TNFα+ IL-2+ Mtb300-specific CD4 T cells exhibited elevated expression of CD27 and lower expression of HLA-DR, KLRG1, and Eomes compared to their CD153− IFNγ+ TNFα+ IL-2+ counterparts (Fig. 5b, left panel). Comparable profiles were observed in aTB participants for CD27 and HLA-DR. But no significant difference in KLRG1 was detected in HIV-infected aTB or in Eomes in HIV-uninfected aTB expression (Fig. 5b, right panel). Overall, these data indicate that CD153-expressing Mtb-specific CD4 T cells exhibit a lower level of differentiation and activation.

### Impact of ATT on CD153 expression by Mtb300-specific CD4 T cells

**Fig. 6:**
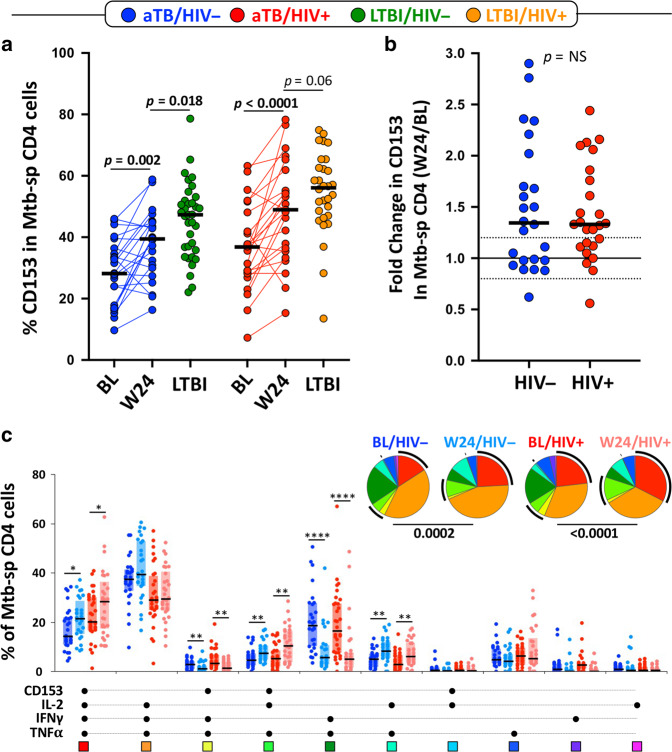
Partial restoration of CD153+ Mtb300-specific CD4 T cells after antitubercular therapy (ATT). **a** Comparison of the proportion of CD153-expressing Mtb300-specific CD4 T cells before (baseline, BL) and after 24 weeks of TB treatment (W24) in HIV- uninfected and HIV-infected patients. Statistical comparisons were performed using a paired nonparametric Wilcoxon test for longitudinal data and a Mann–Whitney test between groups. **b** Fold change in CD153+ Mtb300-specific CD4 T cells between BL and W24. Statistical comparison was performed using a nonparametric Mann–Whitney test. **c** Polyfunctional profile of Mtb300 CD4 responses pre and post ATT. The *x*-axis displays each response pattern, the composition of which is denoted with a dot indicative of the presence of CD153, IL-2, IFNγ, and TNFα. The median (gray bar) and interquartile ranges (box) are shown. Subsets accounting for less than 1% of the total Mtb300-specific CD4 T cell response are not displayed. Each response pattern is color‐coded, and data are summarized in the pie charts. A Wilcoxon rank-sum test was used to compare response pattern between groups (****P < 0.0001, ***P < 0.001, **P < 0.01). Statistical differences between pie charts were defined using a permutation test.

Finally, to define whether ATT restores Mtb-specific CD4 T cell ability to express CD153, we next compared CD153 expression before (i.e., at baseline, BL) and after ATT completion (i.e., at week 24, W24). Despite a significant decrease in the overall frequency of Mtb300-specific CD4 T cells after ATT (median reduction: 42% for HIV− and 61% for HIV+, Supplementary Fig. 4), ATT partially restored CD153 expression in the Mtb-specific CD4 response, irrespective of HIV infection (Fig. 6a). In both HIV-uninfected and HIV-infected patients, the proportion of Mtb300-specific CD4 T cells expressing CD153 increased at W24 compared to BL (*P* = 0.002 and *P* < 0.0001, respectively), but remained lower than values observed in LTBI participants (Fig. 6a). In fact, ATT-associated upregulation of CD153-expressing cells was observed in approximately two-third of the participants (14 out of 24 for HIV-uninfected and 16 out of 25 for HIV-infected participants) (Fig. 6b). Further analysis of the polyfunctional potential of Mtb-specific CD4 T cells show that compared to baseline profiles, Mtb responses post treatment were more polyfunctional with increased proportion of CD153+ IFNγ+ TNFα+ IL-2+ cells, CD153+ IL-2+ TNFα+ cells, and TNFα+ IL-2+ cells, which was counterbalanced by a large contraction of IFNγ+ TNFα+ cells (Fig. 6c).

## Discussion

Our understanding of the immune mechanisms that determine TB disease progression versus containment is still very limited. Identification of potential markers of immune protection against Mtb arises mainly from animal models. As reported in the introduction, two recent publications has highlighted the potential role of CD153 for Mtb protection in a mouse Mtb infection model^7^ and in NHP model of intravenous BCG vaccination.^8^

CD153, a ligand for CD30, is a type II membrane-associated glycoprotein belonging to the TNF super family, which include molecules such as CD70, OX40L, or 4-1BBL. Generally, during antigen recognition, the engagement of these ligands to their cognate receptor will deliver costimulatory signals, via the NF-κ B and stress kinase pathways, leading to the enhancement of survival signaling, cytokine production, and/or cellular proliferation.^16,17^ The role of CD153/CD30 interaction in Mtb infection is still not fully understood. Murine models of Mtb, *M. avium* and BCG infection show that, in contrast to CD70, OX40L, and 4-1BBL, the CD153/CD30 axis may be indispensable for efficient protection against mycobacteria.^7,18,19^

Thus, to define the role of CD153 in TB control in humans, we measured the expression of CD153 on Mtb-specific CD4 T cells in patients classified by HIV and TB status, defined the relationship of CD153 expression with bacterial burden in patients with active TB, and assessed the effect on HIV infection and ATT on this specific cell subset. In this study, we are showing that the proportion of Mtb-specific CD4 T cells expressing CD153 was significantly reduced in active TB patients compared to persons with latent infection (Fig. 2b) and importantly that CD153+ Mtb-specific CD4 response inversely associated with sputum Mtb load inferred by Xpert cycle threshold, irrespective of HIV status (Fig. 4a). To the best of our knowledge, this is the first description of a specific attribute of Mtb-specific CD4 T cells associating with bacterial burden.

Interestingly, we also found an association between Mtb-specific CD4 T cells producing IL-2 and bacterial burden. Based on the capacity of IL-2 to enhance T-cell proliferation, differentiation and survival, it is assumed that it is a key player in the host defense against mycobacteria. However, it remains uncertain whether IL-2 plays a protective role during Mtb infection. As IL-2 and IL-2R knockout mice develop severe autoimmune disease,^20^ such models cannot be used to assess whether IL-2 is required for Mtb protection. Nonetheless, the generation of vaccine-induced IL-2-producing CD4 T cells in mice was associated with enhanced control of Mtb bacterial growth.^21^ In humans, patients with active TB characteristically exhibit decreased proportions of polyfunctional CD4 T cells producing IL-2 in combination with IFNγ and TNFα, compared to latently infected individuals.^22,23^ However, in this study we show that the reduction of polyfunctional CD4 T cells expressing CD153 in aTB patients accounted for most of the contraction of IL-2 producing cells and no significant difference in the proportion of cells coexpressing IFNγ, TNFα, and IL-2 was observed. Therefore, it is possible that the previously described loss of polyfunctional cells during active TB reflects, at least in part, the specific loss of CD153-producing CD4 T cells. The mechanisms underlying CD153-induced protection against Mtb is still unknown. CD30 has been described to be expressed on activated T cells, natural killer (NK) cells, and B cells.^17,24,25^ Thus, it is possible that Mtb-specific CD4 T cells expressing CD153 could (1) provide essential autocrine or paracrine costimulatory signals enhancing CD4 T cell survival and proliferation activity^16,17^ and/or (2) provide help for NK cells to enhance their antibacterial activity. Indeed, several studies demonstrate an important crosstalk between CD4 T cells and NK cells during viral (HIV, CMV, and Flu), fungal, and *Plasmodium falciparum* infection, where antigen-specific CD4 T cell-derived IL-2 enhanced NK responsiveness.^26–31^ Indeed, NK cells have been shown to contribute to Mtb protective immunity, by killing Mtb-infected cells directly or via antibody‐dependent cell‐mediated cytotoxicity.^32,33^

People living with HIV are around 20 times more likely to develop TB^34^ and in high burden countries such as South Africa, HIV-infected persons account for 60% of aTB cases.^35^ While HIV impairs both innate and adaptive immune responses,^5^ the clearest immune defect caused by HIV is a progressive reduction in absolute CD4 T cell numbers that correlates with increasing risk of aTB.^36^ However, shortly after HIV acquisition or when CD4 T cell numbers improve upon antiviral treatment, the risk of aTB remains heightened,^36–38^ suggesting that, in addition to depleting Mtb-specific cells, HIV may also impair their function. Thus, one could speculate that HIV-associated TB risk could be enhanced through alteration of CD153-expressing Mtb-specific CD4 T cells. However, beside the expected reduction of the frequency of Mtb-specific CD4 T cells in HIV-infected persons with LTBI compared to HIV-uninfected individuals (Supplementary Fig. 1 and refs. ^39,40^) we did not observe major alterations in the functional potential of Mtb-specific CD4 T cells in HIV-infected individuals with LTBI compared to uninfected persons and no difference in the proportion of CD153-expressing CD4 T cells were observed. This could be due to the fact that most individuals in the LTBI/HIV+ group were virally suppressed and exhibited relatively well-preserved CD4 counts. It can be also argued that as CD153-expressing CD4 T cells exhibit a low level of memory differentiation and activation (with increased CD27 expression and lower KLRG1, Eomes, and HLA-DR expression compared to their CD153− counterparts, Fig. 5b), these cells could be less susceptible to HIV infection, as HIV preferentially targets activated cells.^41,42^

Further mechanistic studies are required to investigate whether Mtb-specific CD4 T cells expressing CD153 is a cause or a consequence of reduced bacterial burden. Moreover, it remains to be determined if the distribution of Mtb-specific CD4 T cells expressing CD153 observed in blood is comparable to the site of disease. However, building on evidence from animal models, our results underline the key potential role of CD153 in containment of Mtb in humans. With promising new TB vaccine approaches such as M72/AS01E^43^ coming to light, our data also provide a strong rationale to include the measurement of CD153 in Mtb-specific CD4 T cells to evaluate novel TB vaccine candidates.

## Methods

### Study population

Participants were recruited from the Ubuntu Clinic, Site B, Khayelitsha (Cape Town, South Africa) between March 2017 and December 2018. All participants were adults (age ≥ 18 year) and provided written informed consent. The study was approved by the University of Cape Town Human Research Ethics Committee (HREC 050/2015) and was conducted under DMID protocol no. 15–0047. Those in the active TB group (*n* = 70) all tested sputum Xpert MTB/RIF (Xpert, Cepheid) positive. All active TB cases were drug sensitive and had received no more than one dose of ATT at the time of baseline blood sampling. The latent TB healthy control group (*n* = 68) were all asymptomatic, had a positive IFNγ release assay (IGRA, QuantiFERON®-TB Gold In-Tube), tested sputum Xpert MTB/RIF negative and had no clinical evidence of active TB. Sputum Xpert MTB/RIF, sputum culture for Mtb, CD4 count, HIV VL, and CRP tests were performed by the National Health Laboratory Services. Clinical characteristics of the study participants can be found in Table 1.

### Timika scoring of chest radiographs

Chest radiographs from the enrollment visit of those with active TB were scored by the study clinician using the Timika score.^44^ Previously published user guidelines were strictly adhered to.^45^ Briefly, posteroanterior chest radiographs were assessed for the total percentage of the lung fields affected by known features of active pulmonary TB. A value of 40 was added to the overall percentage affected lung where at least one cavity ≥1 cm could be identified.

### Blood collection and whole blood assay

Blood was collected in sodium heparin tubes and processed within 3 h of collection. The whole blood assay was adapted from the protocol described by Hanekom et al.^46^ Briefly, 0.5 ml of whole blood was stimulated with a pool of 300 Mtb-derived peptides (Mtb300, 2 µg/ml)^47^ at 37 °C for 5 h in the presence of the costimulatory antibodies, anti-CD28 and anti-CD49d (1 μg/ml each; BD Biosciences) and Brefeldin-A (10 µg/ml; Sigma-Aldrich). Unstimulated cells were incubated with costimulatory antibodies and Brefeldin-A only. Red blood cells were then lysed in a 150 mM NH_4_Cl, 10 mM KHCO_3_, and 1 mM Na_4_EDTA solution. Cells were stained with a Live/Dead Near-InfraRed dye (Invitrogen), and then fixed using a Transcription Factor Fixation buffer (eBioscience), cryopreserved in freezing media (50% fetal bovine serum, 40% RPMI, and 10% dimethyl sulfoxide) and stored in liquid nitrogen until use.

### Cell staining and flow cytometry

Cryopreserved cells were thawed, washed and permeabilized with a Transcription Factor perm/wash buffer (eBioscience). Cells were then stained at room temperature for 45 min with the following antibodies: CD3 BV650 (OKT3; Biolegend), CD4 BV785 (OKT4; Biolegend), CD8 BV510 (RPA-T8; Biolegend), CD27 PE-Cy5 (1A4CD27; Beckman Coulter), HLA-DR BV605 (L243; Biolegend), Killer cell Lectin-like Receptor G1 (KLRG1) PerCP-eFluor 710 (13F12F2, eBioscience), Eomes eFluor 660 (WD1928, eBioscience), IFNγ BV711 (4S.B3; Biolegend), TNFα eFluor 450 (Mab11; Biolegend), IL-2 PE/Dazzle (MQ1-17H12, Biolegend), and CD153 (R&D116614, R&D). Samples were acquired on a BD LSR-II and analyzed using FlowJo (v9.9.6, TreeStar). A positive cytokine response was defined as at least twice the background of unstimulated cells. To define the phenotype of Mtb300-specific cells, a cut-off of 30 events was used. The gating strategy is presented in Supplementary Fig. 5.

### Statistical analyses

Statistical tests were performed in Prism (v8.2; GraphPad). Nonparametric tests were used for all comparisons. The Kruskal–Wallis test with Dunn’s Multiple Comparison test was used for multiple comparisons and the Mann–Whitney and Wilcoxon matched pairs test for unmatched and paired samples, respectively.

## Supplementary information

Supplementary Information
