# Supplementary material for: Mycobacterium tuberculosis-specific CD4 T cells expressing CD153 inversely associate with bacterial load and disease severity in human tuberculosis
Source: Mucosal Immunol. 2020 Jul 16;14(2):491–9. doi: 10.1038/s41385-020-0322-6 (PMC7855386; doi:10.1038/s41385-020-0322-6)
Supplement: Supplementary file 1 — Supplementary Information [file 41385_2020_322_MOESM1_ESM.pdf]

Supp Figure 1

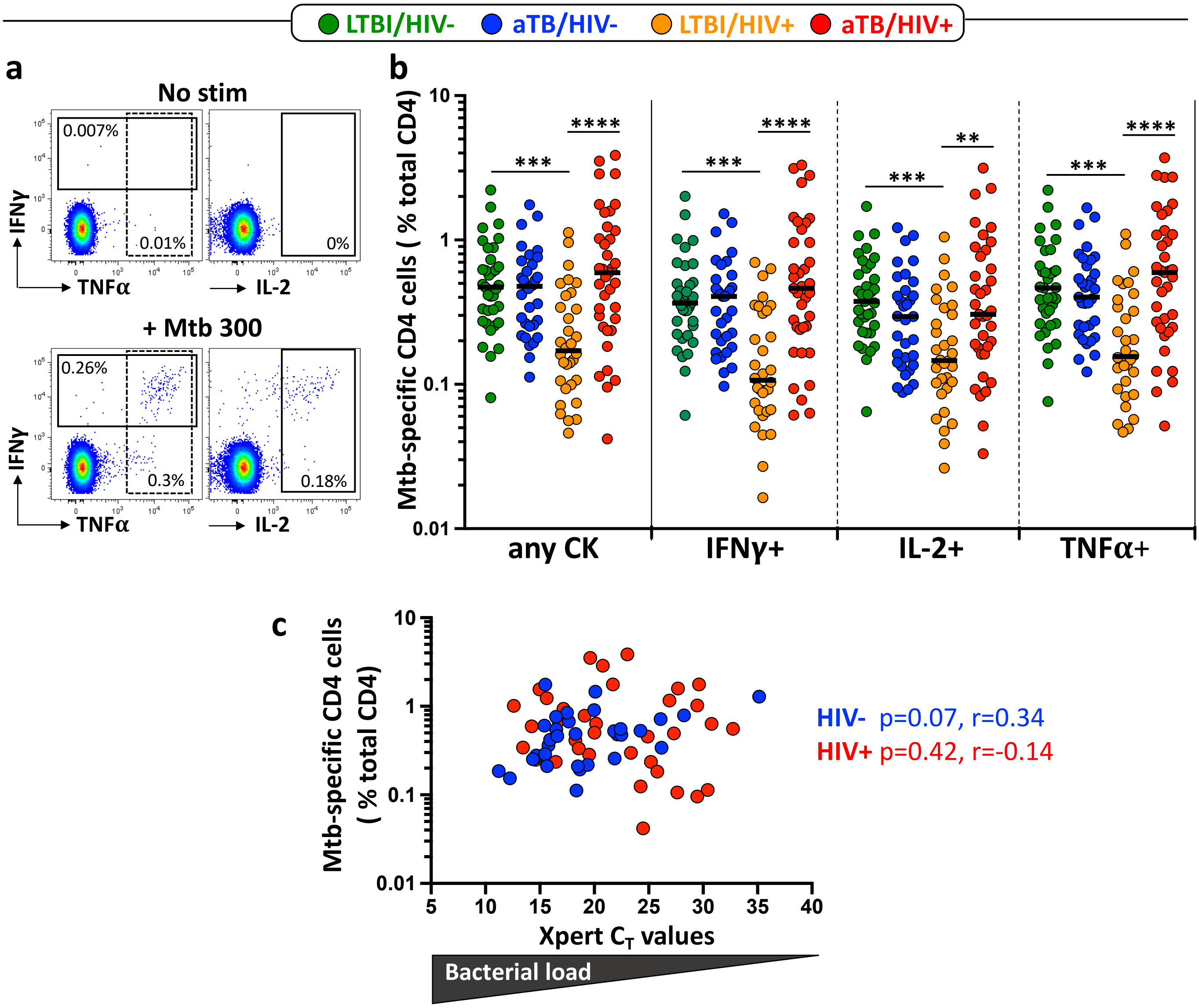

Supplementary Figure 1. Magnitude of Mtb300-specific CD4 T cell responses in the four study groups. a Representative flow plots of IL2, IFNγ and TNFα expression in response to Mtb300 in CD4 T cells. No stim = no stimulation. **b** Summary graph of the magnitude of Mtb300-specific CD4 T cells producing any measured cytokine, IFNγ, IL2 or TNFα in LTBI/HIV- (n = 35, green), aTB/HIV- (n = 33, blue), LTBI/HIV+ (n = 33, orange) and aTB/ HIV+ (n = 37, red). Medians are depicted. Statistical comparisons were performed using One-way ANOVA Kruskal-Wallis test with a Dunn's multiple comparison test. **c** Correlation between Xpert C_T values and the magnitude of Mtb300-specific CD4 T cells (producing any cytokines) in HIV-uninfected and HIV-infected aTB patients. Correlations were tested by a two-tailed non-parametric Spearman rank test.

Supp Figure 2

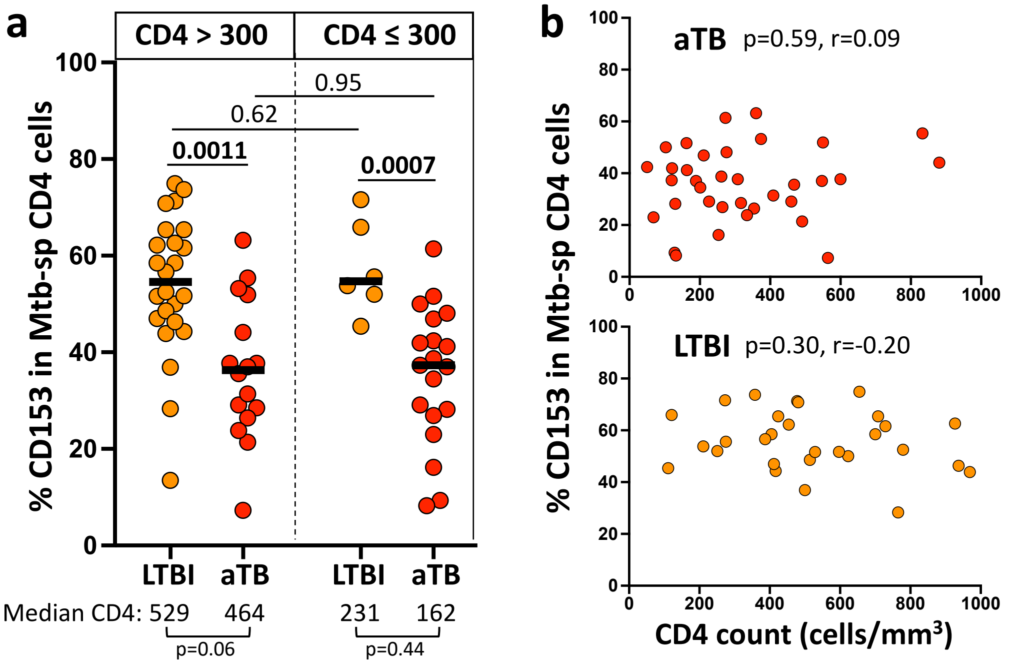

Supplementary Figure 2. a CD153 expression in Mtb300-specific CD4 T cells in LTBI and aTB HIV-infected participants stratified based on absolute CD4 count (i.e CD4 count > 300 cells/mm^3 and CD4 count ≤ 300 cells/mm^3). Median CD4 count for each sub-group is presented at the bottom of the graph. Statistical comparisons were performed using a Mann-Whitney test. **b** Correlation between CD4 count and the proportion of CD153+ Mtb300-specific CD4 T cells in the aTB (top) and LTBI (bottom) groups. Correlations were tested by a two-tailed non-parametric Spearman rank test.

Supp Figure 3

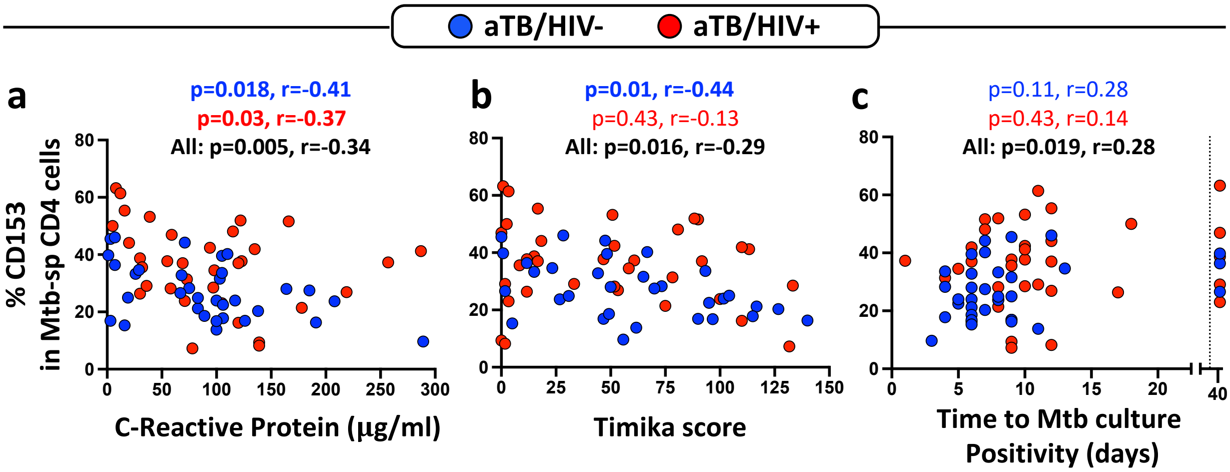

Supplementary Figure 3. Relationship between the proportion of Mtb300-specific CD4 T cells expressing CD153 and **a** plasma C-reactive protein, **b** Timika score and **c** time to Mtb culture positivity in HIV-uninfected (blue) and HIV-infected (red) participants with aTB. Correlations between measures were performed using a two-tailed non-parametric Spearman test.

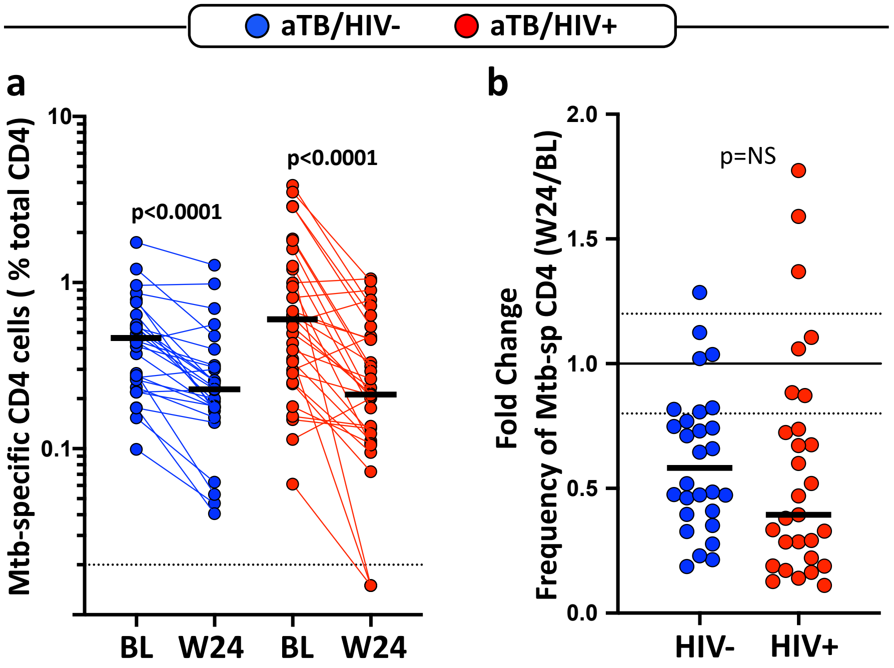

Supplementary Figure 4. Evolution of the magnitude of Mtb300-specific CD4 T cell responses pre- (baseline, BL) and post-TB treatment (week 24, W24). ***a*** Frequency of Mtb300-specific CD4+ T cells at BL and Week 24 post-TB therapy. Medians are depicted. Values below the dashed line correspond to undetectable Mtb300 responses. Statistical comparisons were performed using a paired, non-parametric Wilcoxon test. **b** Fold change in the frequency of Mtb300-specific CD4 T cells between BL and W24. Statistical comparison was performed using a non-parametric Mann-Whitney test.

Supp Figure 5

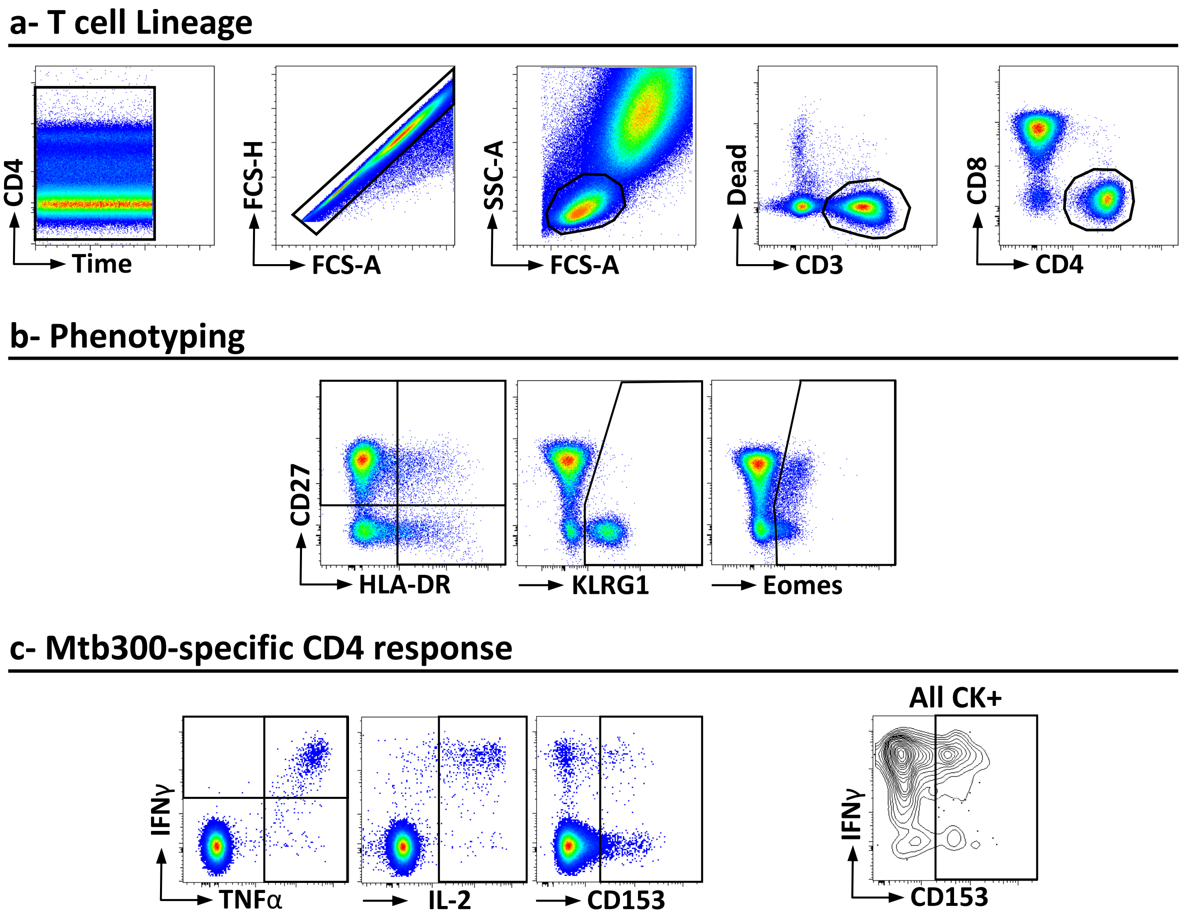

Supplementary Figure 5. Gating strategy. a Gating strategy for the identification of CD4 T cells. Doublets and dead cells were excluded from the analysis. **b** Gating strategy applied for phenotypic markers (CD27, HLA-DR, KLRG1 and Eomes). Plots represented are gated on CD4+ T cells. **c** Gating strategy for IL2, IFNγ, TNFα and CD153 expression in response to Mtb300 in CD4 T cells. The contour plot on represents IFNγ and CD153 expression in Mtb300-specific CD4 T cells expressing any of the measured cytokines (All CK+: IL-2, IFNγ or TNFα).
